# Factors Associated with Vascular Changes at the Level of Retinal Ganglion Cell Axon versus Soma/Dendrite in Glaucoma Patients

**DOI:** 10.3390/jcm12134221

**Published:** 2023-06-22

**Authors:** Si-Eun Oh, Hee-Jong Shin, Chan-Kee Park, Hae-Young Lopilly Park

**Affiliations:** Department of Ophthalmology, Seoul St. Mary’s Hospital, College of Medicine, The Catholic University of Korea, Seoul 06591, Republic of Korea

**Keywords:** glaucoma, optical coherence tomography angiography, vessel density

## Abstract

Superficial and deep macular vessel density (VD) is decreased in eyes with glaucoma. Superficial VD comprises both the retinal nerve fiber layer (RNFL) and ganglion cell/inner plexiform layer (GC/IPL), and various terms have been used previously to describe the layers of macular VD. In our study, we readjusted the macular segmentation. We obtained RNFL and GC/IPL VDs separately to evaluate VD changes of axon versus soma/dendrite of the retinal ganglion cells (RGCs) in detail. We included 66 eyes of normal tension glaucoma patients with inferior localized RNFL defects solely impacting the inferior hemiretina. Macular VD was measured as RNFL VD and GC/IPL VD. VD ratio was calculated by dividing the VD from the affected hemiretina by the VD from the unaffected hemiretina. RNFL VD ratio was related to RNFL and GC/IPL thicknesses (*p =* 0.005, *p =* 0.001), whereas GC/IPL VD ratio was not (*p =* 0.596, *p =* 0.783). A lower GC/IPL VD ratio was associated with lower RNFL VD (*p =* 0.017) and systemic hypertension (*p =* 0.03) in multivariate analysis. Patients with a reduced GC/IPL VD ratio were more prone to poor visual field defects (*p =* 0.022) and paracentral scotoma (*p =* 0.046) and more likely to be on treatment for systemic hypertension (*p =* 0.024). Therefore, glaucoma patients on systemic hypertension treatment and reduced GC/IPL VD require cautious management.

## 1. Introduction

Glaucoma is pathologically characterized by retinal ganglion cells (RGCs) loss. The optic nerve head (ONH) is thought to be the primary site of glaucoma pathology [1,2,3]. Numerous studies have focused on the ONH, the unique structure where axons of the RGCs from all points of the retina converge together and bend sharply to exit the globe through the lamina cribrosa [4,5,6]. RGC is a tripartite structure with soma located in the ganglion cell layer (GCL), dendrites in the inner plexiform layer (IPL), and long axons through the retinal nerve fiber layer (RNFL) projecting to the lateral geniculate nucleus [3,7,8,9]. Each compartment of the RGC resides in different layers and locations in the eye, so the vascular supply within a single cell is nonuniform.

The importance of ocular blood flow in glaucoma has been emphasized in numerous studies. With advances in optical coherence tomography angiography (OCTA), ocular blood flow has been quantified as vessel density (VD) at various locations within the eye, including the ONH, peripapillary (PP), and macular region, and obtaining the VD of a specific layer has become possible. A reduction in VD results from the loss of vessels within the inner retinal tissues, along with a loss of RGCs. However, electrophysiological studies have shown that vessel loss may precede RGC loss or VD reduction at the stage of RGC dysfunction in glaucoma suspects or early pre-perimetric glaucoma [10]. Therefore, VD measurement using OCTA can be used to determine the functional status of RGCs.

Macular VD is associated with glaucoma. A decrease in superficial and deep macular VDs is observed in glaucomatous eyes compared to healthy and glaucoma-suspect eyes [11]. A correlation exists between the superficial macular VD and the thickness of RNFL and ganglion cell (GC)/IPL. Additionally, deep macular VD is related to visual field (VF) progression and paracentral scotoma, independent of structural parameters [12,13]. However, various terms have been used to describe the layers of macular VD, which are not uniform, as different OCTA devices have been used (AngioVue OCTA -Optovue Inc. Fremont, CA, USA; AngioPlex–Zeiss Meditec, Inc., Dublin, CA, USA; DRI OCT Triton, Topcon, Tokyo, Japan) to evaluate macular VD changes in many studies [13,14,15,16,17,18,19,20,21]. Automated layer segmentation applied to OCT instrument software has shown the superficial macular VD comprising mainly of RNFL, GCL, and IPL from the internal limiting membrane (ILM) to the inner border of the inner nuclear layer (INL). Deep macular VD comprises the INL from the inner to outer borders where the bipolar cells are located.

We aimed to determine the factors and functional outcomes associated with the vascular changes of each compartment of RGCs in the pathology of glaucoma. Hence, we evaluated the VD changes in the soma and dendrites (GC/IPL VD), axons in the macula (RNFL VD), and axons around the ONH (PP VD) of RGCs

## 2. Materials and Methods

### 2.1. Participants

Sixty-six eyes of normal tension glaucoma (NTG) patients with localized RNFL defects in the inferior hemiretina who only visited the glaucoma clinic of Seoul St. Mary’s Hospital between January 2022 and September 2022 were included in the study.

As described in previous studies, NTG was defined as intraocular pressure (IOP) < 21 mmHg without the use of IOP-lowering eye drops at the first clinic visit, accompanied by the presence of open-angle and glaucomatous optic nerve damage [22,23]: diffuse or localized rim thinning, disc hemorrhage, a notch in the rim, a vertical cup-to-disc ratio higher than that of the other eye by more than 0.2, or compatible repeated VF damage. Glaucomatous VF defects were defined according to the criteria used in previous studies [12,24] as follows: (1) glaucoma hemifield test results outside normal limits, (2) ≥three adjacent points with a probability of <5% of the normal population, with one of these points having a probability of <1%, or (3) a pattern standard deviation (PSD) with a *p*-value < 5%.

The inclusion criteria were as follows: (1) A best corrected visual acuity of 20/40 or better, (2) a localized wedge-shaped RNFL defect in the inferior hemiretina detectable on red-free RNFL photography, (3) asymptomatic to mild glaucomatous VF loss (mean deviation [MD] > −6 dB), (4) age > 18 years, (5) open angles on gonioscopy, and (6) well-controlled disease status with IOP-lowering eye drops. Patients who had undergone intraocular surgery other than cataract surgery were excluded. Moreover, patients with known retinal diseases were also excluded because of their possible impact on macular VD measurements.

Paracentral scotoma was defined as VF defects on a pattern deviation probability map, with clusters of three or more test points with a <5% probability or two or more points with a ≤1% probability within the 12 points of central 10° [13].

Microvascular dropout was defined according to the criteria used in a previous study [25] as focal and sectoral choriocapillary dropout, with a width greater than twice that of the visible juxtapapillary microvessels observed on the parapapillary choroidal microvasculature map obtained from OCTA.

On the first day of the visit, all participants underwent ophthalmic examinations, including slit-lamp examination, Goldmann applanation tonometry, central corneal thickness (UD-800; Tomey Corporation, Nagoya, Japan), red-free fundus photography (Canon; Tokyo, Japan), RNFL and GC/IPL thickness measurement using OCT (DRI OCT Triton; Topcon, Tokyo, Japan), and Humphrey VF examination using the Swedish Interactive Threshold Standard (SITA) 24-2 algorithm (Carl Zeiss Meditec, Inc., Dublin, CA, USA). All patients underwent OCTA (DRI OCT Triton, Topcon, Tokyo, Japan) for VD measurement during the follow-up period. Detailed assessments of patient history and review of medical/ocular records and prescriptions were performed to determine the history of systemic hypertension and diabetes mellitus. All disc hemorrhages that occurred during follow-up were recorded. The mean IOP was the average of the measurements obtained during the last three visits.

### 2.2. OCTA and VD Measurement

**Figure 1:**
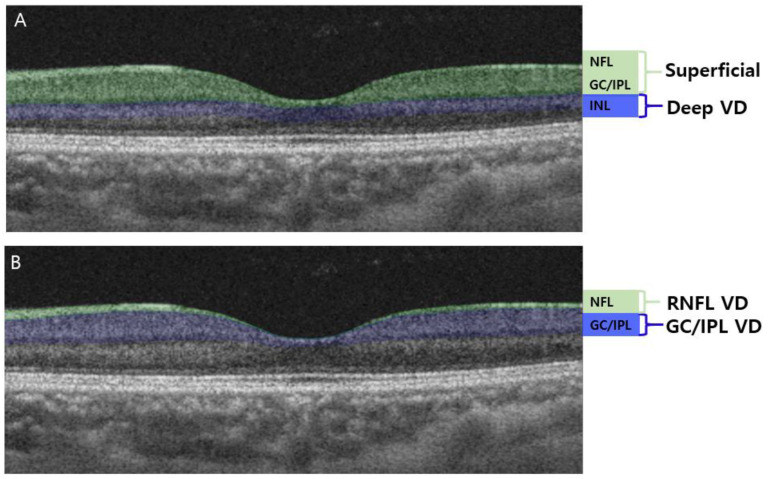
Optical coherence tomography angiography (OCTA) segmentation of macular vessel density (VD). (**A**) OCTA segmentation in previous studies: superficial macular VD comprised the retinal nerve fiber layer (RNFL), ganglion cell layer (GCL), and inner plexiform layer (IPL). The deep macular VD comprised the inner nuclear layer (INL). (**B**) OCTA segmentation readjusted in this study: RNFL VD comprised only the RNFL layer of the superficial vasculature, whereas the GC/IPL VD comprised the GCL and IPL.

A Swept-source OCTA device with a scanning speed of 100,000 A-scans per second using a wavelength of 1050 nm was used to measure the vascular status of the ONH and macula. Scans of 4.5 × 4.5 mm were obtained. The PP and RNFL VD extended from the ILM to the RNFL. The GC/IPL VD extended from the outer border of the RNFL to the inner border of the INL. This segmentation was different from that used in our previous studies [13,22,26], where the superficial vasculature extended from the ILM to the inner border of the IPL, and the deep vasculature extended from the inner border of the IPL to the outer border of the INL (Figure 1A). To identify VD changes of the RNFL and GC/IPL, the axon versus soma/dendrite of the RGCs, the layers of OCTA were readjusted, and VD measurements were obtained from the RNFL and GC/IPL separately (Figure 1B).

**Figure 2:**
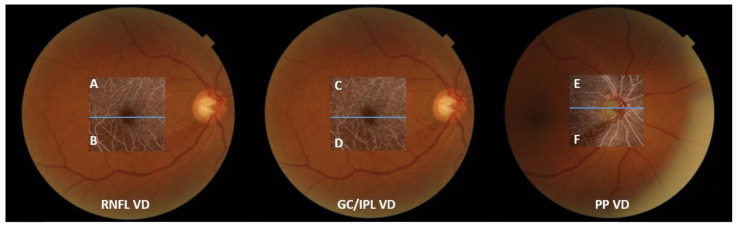
An illustrative description of obtaining vessel density (VD) ratio, with fundus photos of a participant with an inferior retinal nerve fiber layer (RNFL) defect. From the left are optical coherence tomography angiography images of the RNFL VD, ganglion cell/inner plexiform layer (GC/IPL) VD, and peripapillary (PP) VD. The macular and PP areas were divided according to the center of the fovea and disc (blue lines). RNFL and GC/IPL VDs were calculated as the ratio of VD of the inferior hemiretina and the unaffected superior hemiretina, i.e., “B/A” and “D/C.” PP VD ratio was calculated as “F/E.” The ratio between the RNFL VD and PP VD at the affected hemiretina was calculated as “B/F”, and the ratio between the GC/IPL VD and PP VD was calculated as “D/F”.

VD measurements from OCTA images were performed as described in previous studies [24,27]. Only the images with a quality score >60 were selected. Eyes with poor image quality due to blurring, fixation, and macular segmentation errors were excluded. OCTA measurements were not performed if a disc hemorrhage occurred that day due to the possible influence of the disc hemorrhage on the PP VD image quality. The mean threshold algorithm was used to create an 8-bit binarized image. As the white pixels represented “vessel” and black pixels represented “background”, the VD was calculated as the percentage of the white pixel area divided by the total image area. VDs at the superior and inferior hemiretina were also measured separately at the optic disc and macular regions. These measurements were divided according to the center of the disc for peripapillary VD and the center of the fovea for macular VD. The VD ratio was defined as the ratio of the impaired half of the VD divided by the VD of the less affected side to show the degree of decompensation of the impaired half. As all 66 eyes included in this study had inferior RNFL defects, the ratio was calculated as the ratio of the inferior to superior VD. The ratios between the RNFL VD and PP VD, GC/IPL VD, and PP VD of the inferior hemiretina were calculated to determine the factors associated with changes in the RNFL and GC/IPL VDs, not as a result of decreased PP (Figure 2).

### 2.3. Statistical Analysis

All values were presented as means ± standard deviations. Student’s *t*-test and the chi-square test were used to compare variables between the higher and lower macular VD ratio groups. Pearson correlation analysis determined factors correlated with RNFL and GCIPL VD ratio. Univariate and multivariate linear regression analyses were used to identify the factors associated with various macular VD ratios. All statistical analyses were performed using SPSS (version 24.0; SPSS Inc., Chicago, IL, USA). Statistical significance was set at *p* < 0.05.

## 3. Results

We included 66 eyes of NTG patients with inferior localized RNFL defects. The demographic features of the participants are presented in Table 1. Patients had a mean age of 53.73 ± 12.22 years and average MD of the VF as −2.39 ± 1.99 dB. The mean RNFL VD was 40.92 ± 1.26% and the mean GC/IPL VD was 44.80 ± 1.64%. We measured the GC/IPL VD ratio and sorted the eyes into two groups: 33 eyes (50%) with a high ratio of GC/IPL VD and 33 eyes (50%) with a low ratio of GC/IPL VD. The low-ratio group represented eyes with greater VD reduction in the inferior GC/IPL, where the localized RNFL defect was located, compared to the unaffected superior hemiretina. The low GC/IPL-ratio group had a lower ratio of PP VD (*p* = 0.015), a lower ratio of RNFL VD (*p* < 0.001), worse VF parameters (lower MD [*p* = 0.022] and higher PSD [*p =* 0.035]). It was more likely to be on treatment for systemic hypertension (*p =* 0.024) and also exhibited paracentral scotoma (*p =* 0.046). There was no significant difference between the groups with respect to the prevalence of diabetes mellitus (Table 2).

We also evaluated the correlations between the RNFL and GC/IPL VD ratios and other ocular parameters (Table 3). Both RNFL and GC/IPL VD showed a significant correlation with the PP VD ratio and MD of VF. The RNFL VD ratio was significantly correlated with RNFL (R = 0.343, *p =* 0.005) and GC/IPL thickness (R = 0.409, *p =* 0.001; Table 3); however, its correlation with GC/IPL VD was not significant (R = 0.067, *p =* 0.596 and R = 0.034, *p =* 0.783, respectively).

Regression analyses were performed to determine the factors associated with the GC/IPL VD ratio (Table 4). In the univariate analysis, a lower GC/IPL VD ratio was associated with a lower RNFL VD (β = 0.34, 95% confidence interval [CI] = 0.013–0.072, *p =* 0.005), lower MD of the VF (β = 0.019, 95% CI = 0.000–0.038, *p =* 0.049), and more frequent treatment for systemic hypertension (β = −0.302, 95% CI = −0.260–−0.031, *p =* 0.014). Whereas, in the multivariate analysis, a lower GC/IPL VD ratio was associated with a lower RNFL VD (β = 0.302, 95% CI = 0.007–0.069, *p =* 0.017) and more frequent treatment for systemic hypertension (β = −0.257, 95% CI = −0.235–−0.012, *p =* 0.03).

Further analysis was conducted to determine the significance of changes in the GC/IPL VD independent of a decreased PP VD or RNFL VD (Table 5 and Table 6). The ratio between the RNFL VD and PP VD at the affected hemiretina was calculated, and regression analysis was performed (Table 5). In the univariate analysis, GC/IPL thickness (β = 0.283, 95% CI = 0.001–0.010, *p =* 0.021), PSD of the VF (β = −0.298, 95% CI = −0.021 to −0.002, *p =* 0.015), and the presence of paracentral scotoma (β = −0.564, 95% CI = −0.171 to −0.079, *p* < 0.001) showed significant associations. In the multivariate analysis, the presence of paracentral scotoma (β = −0.115, 95% CI = −0.167 to −0.064, *p* < 0.001) was the only associated factor.

These results indicated that the damage location in the macular region was associated with the RNFL VD more than it was associated with PP VD; therefore, the ratio between the GC/IPL VD and PP VD in the affected hemiretina was calculated and analyzed (Table 6). A lower ratio between the macular GC/IPL VD and PP VD in the affected retina was associated with systemic hypertension treatment (β = −0.138, 95% CI = −0.211 to −0.055, *p =* 0.001).

**Figure 3:**
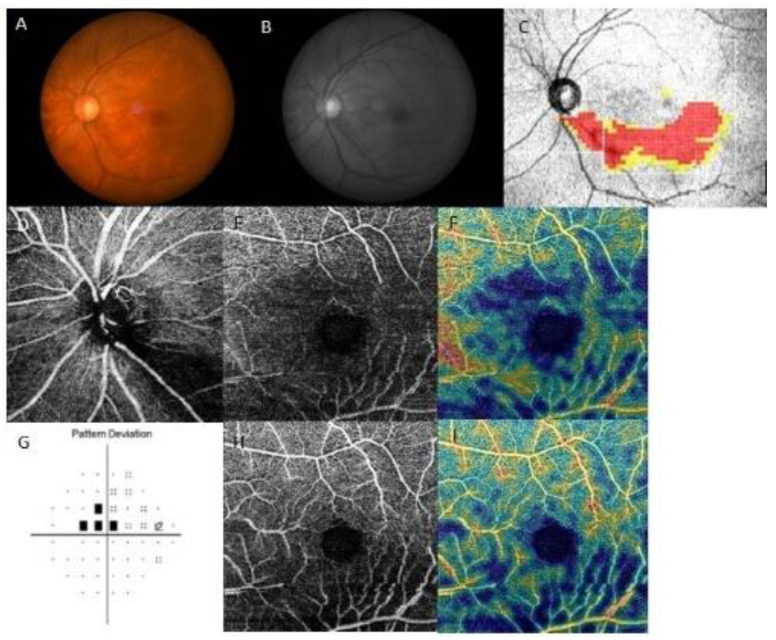
A representative case of a 67-year-old female on systemic hypertension treatment with paracentral scotoma. The left eye had an inferior retinal nerve fiber layer (RNFL) defect, and a decrease in RNFL and ganglion cell/inner plexiform layer (GC/IPL) vessel density (VD) was seen at the inferior hemiretina. (**A**) Fundus photography and (**B**) red-free photography showing localized, inferotemporal RNFL defect in the left eye. (**C**) Optical coherence tomography (OCT) GCL++ (RNFL + GCL + IPL) thickness map showing the inferotemporal RNFL defect. OCT angiography of (**D**) peripapillary VD, (**E**) RNFL VD, and (**H**) GC/IPL VD. Density maps of (**F**) RNFL and (**I**) GC/IPL VD visualizing the marked decrease in both RNFL and GC/IPL VD. (**G**) Pattern deviation probability map of Swedish Interactive Threshold Standard 24-2 showing paracentral scotoma.

**Figure 4:**
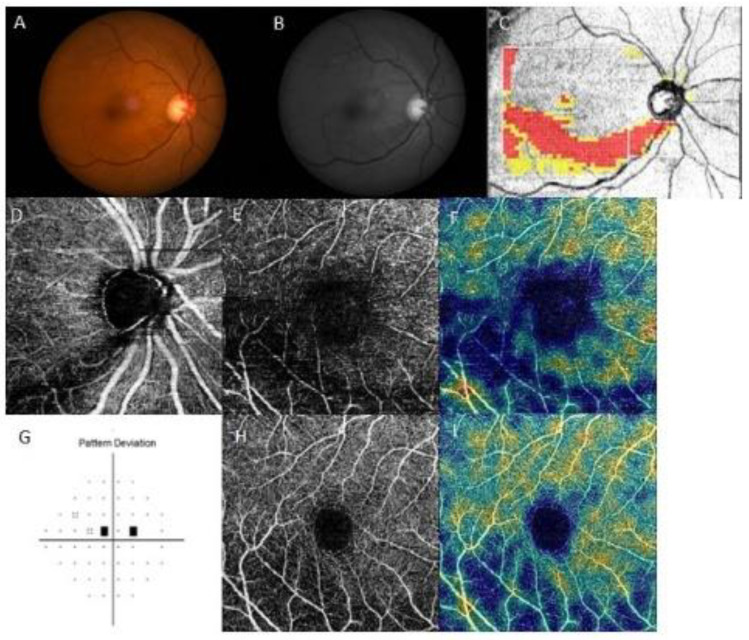
A representative case of a 64-year-old female without systemic hypertension and paracentral scotoma. The right eye had an inferior retinal nerve fiber layer (RNFL) defect, and RNFL vessel density (VD) was decreased, whereas the ganglion cell/inner plexiform layer (GC/IPL) VD was relatively preserved compared to that in the representative case in Figure 3. (**A**) Fundus photography and (**B**) red-free photography showing a localized, inferotemporal RNFL defect in the right eye. (**C**) Optical coherence tomography (OCT) GCL++ (RNFL + GCL + IPL) thickness map showing the inferotemporal RNFL defect. OCT angiography of (**D**) peripapillary VD, (**E**) RNFL VD, and (**H**) GC/IPL VD. Density maps of (**F**) RNFL and (**I**) GC/IPL VD showed a marked decrease in RNFL VD, whereas the GC/IPL VD was relatively preserved. (**G**) Pattern deviation probability map of Swedish Interactive Threshold Standard 24-2 showing paracentral scotoma.

Representative cases are shown in Figure 3 and Figure 4. Both patients had inferior RNFL defects with similar VF damage in the central region. However, patients on treatment for systemic hypertension (Figure 3) had a more impaired GC/IPL VD than patients without systemic hypertension (Figure 4), relative to the similar RNFL VD loss.

## 4. Discussion

Analysis of VD of the superficial capillary plexus of the macula and PP region has been the subject of previous studies [28,29,30]. In this study, we applied a novel approach to further differentiate the VD of the superficial capillary plexus into VD related to the axon and soma/dendrite of the RGCs as the RNFL and GC/IPL VD.

We demonstrated an association between impaired GC/IPL VD and systemic hypertension in NTG patients. The decrease in RNFL VD was related to structural deterioration and thinning of the RNFL and GC/IPL due to glaucomatous damage. This agrees with previous studies [12,28,29,30] that measured superficial VD as vessels within the RNFL, GCL, and IPL. The GC/IPL VD was associated with PP or macular RNFL VD changes. However, our study aimed to identify factors associated with changes in VD within the GCL and IPL, independent of changes within the PP area or macular RNFL. Our findings suggest GC/IPL VD indicates local ocular blood circulation independent of the glaucomatous structural damage. To our knowledge, this is the first study to identify the impact of systemic hypertension on the decrease in GC/IPL VD in NTG patients.

The radial peripapillary capillary plexus (RPCP) supplies the NFL, which is dense in the PP region [31]. Studies have evaluated the extent of RPCP as PP VD and RNFL VD of the macula. As the RPCP runs parallel to the NFL axons, there was a significant decrease in PP VD where the RNFL defect was located. The RNFL VD was also greatly affected by RNFL and GC/IPL thinning. However, the GC/IPL vascular plexus measured in this study was mainly located in the GCL and IPL, and the GC/IPL VD did not significantly correlate with structural parameters related to glaucomatous damage, such as the RNFL and GC/IPL thicknesses.

Furthermore, GC/IPL VD was significantly associated with systemic hypertension, independent of the RNFL VD. The GCL and IPL are the layers where the soma and dendrites of RGCs are located. These layers may be vulnerable to blood flow instability due to systemic hypertension because they are located at the junction of the inner retinal blood supply and choroidal blood supply. Additionally, soma/dendritic regions are known to have high metabolic demands [3]. Therefore, these RGC regions may be more susceptible to vascular insufficiency. Axonal damage is the primary source of damage in glaucoma; however, our findings indicate that early soma/dendrite functional changes can occur in eyes with systemic hypertension.

Penteado et al. [32] evaluated superficial macular VD measured from 3 µm below the ILM to 15 µm below the IPL and reported that superficial macular VD was associated with central VF loss. Jeon et al. [13] reported that deep macular VD, measured from 15.6 µm below the IPL/INL to 70.2 µm below the IPL/INL, was related to central visual function and VF progression. Deep macular VD was an independent factor affecting visual function and unrelated to structural parameters. The superficial macular VD evaluated in previous studies included the RPCP and superficial vascular plexus, comprising vessels within the NFL, GCL, and IPL, while the deep macular VD comprised the intermediate capillary plexus and some of the deep capillary plexus [31]. Since the superficial VD included vessels of not just the RNFL but also of the GCL and IPL, the importance of the VD of the GC/IPL was often underestimated due to the secondary VD decrease caused by structural thinning. We readjusted the OCTA layer segmentation to finely evaluate the vasculature of different parts of the RGC separately, as in RNFL and GC/IPL VD. The RNFL VD included vessels comprising only the RNFL, and vessels supplying the GCL and IPL were included in the GC/IPL VD. Therefore, the GC/IPL VD in this study focused on the vasculature of the RGC soma and the synapses of the dendrites, which were unaffected by RNFL thinning. By adjusting the OCTA parameters, we identified factors associated with VD changes within the RGC axons versus soma/dendrite compartments.

GC/IPL VD represents the blood supply of retinal layers where the soma of RGCs reside, and the dendrites synapse with the bipolar and amacrine cells [3]. The Synapse is an energy-demanding component of neurons [33], and this is also seen in the retina as the outer region of the IPL has a low oxygen tension owing to the high oxygen consumption rate [34]. This study investigated ocular blood flow that affects the soma and dendrites of RGCs. In addition to the blood flow of the ONH, the deep macular blood flow of soma and dendrites of RGCs also has an impact on glaucoma. Moreover, systemic hypertension is an essential factor associated with it.

The association between systemic hypertension and glaucoma has been investigated extensively. Systemic hypertension influences elevation in IOP [35]; however, the Blue Mountains Eye Study [36] showed that hypertension is related to an increased risk of open-angle glaucoma, independent of the effect of blood pressure on IOP. As seen in our study, the GC/IPL VD decrease and systemic hypertension were strongly associated factors, and the GC/IPL VD may be a possible connecting link of hypertension in the pathophysiology of glaucoma.

The present study had several limitations. First, we evaluated only NTG patients. As these patients are known to have more vascular abnormalities [26,37], the VD assessment in this study could not be generalized to all types of glaucoma. Second, the presence of projection artifacts and errors in the automatic segmentation of OCTA could be another limitation which may impact the accuracy of the VDs obtained using OCTA, considering they were important ocular parameters in this study. We attempted to overcome this limitation by including images with a quality score over 60 and excluding images of low quality. Third, since the VD parameters had very small differences in the numerical values, the ratios of various parameters were used to show the VD impairment and factors associated with VD changes. Further studies are needed to determine whether systemic hypertension or its treatment drives the VD changes, and the class of antihypertensive medications used by the patients can be further analyzed.

In summary, by readjusting the OCTA segmentation into RNFL and GC/IPL VD, we have demonstrated that glaucoma patients with vascular insufficiency within the GC/IPL were more likely to be on treatment for systemic hypertension. Patients with reduced GC/IPL VD were more likely to have worse VF defects and paracentral scotoma. These findings were independent of the RNFL and GC/IPL structures, the main structural parameters associated with the progression of glaucoma. This indicates that VD changes within the GC/IPL are independent of the glaucomatous process or result from axonal damage but may reflect the functional status of the soma/dendrites of the RGCs. Glaucoma patients on systemic hypertension treatment and reduced GC/IPL VD require careful management, as they are more likely to have worse VF defects and paracentral scotoma.

## Figures and Tables

**Table 1:** Baseline characteristics of participants.

Variables	66 Eyes
Age, years	53.73 ± 12.22
Sex, male/female	23/43
BCVA, decimals	0.94 ± 0.12
Baseline IOP, mmHg	15.33 ± 2.51
Mean IOP, mmHg	14.01 ± 2.67
CCT, µm	534.29 ± 35.38
Spherical equivalent, D	−2.06 ± 2.64
RNFL thickness, µm	89.94 ± 11.17
GC/IPL thickness, µm	63.36 ± 5.51
PP VD	45.56 ± 1.30
RNFL VD (%)	40.92 ± 1.26
GC/IPL VD (%)	44.80 ± 1.64
Disc hemorrhage, *n* (%)	16 (24.2%)
Microvasculature dropout, *n* (%)	30 (46.2%)
MD, dB	−2.39 ± 1.99
PSD, dB	4.37 ± 2.85
Paracentral scotoma, *n* (%)	38 (57.6%)
Diabetes mellitus, *n* (%)	6 (9.1%)
Systemic hypertension, *n* (%)	8 (12.1%)

BCVA = best corrected visual acuity; IOP = intraocular pressure; CCT = central cornea thickness; D = diopter; RNFL = retinal nerve fiber layer; GC/IPL = ganglion cell/inner plexiform layer; PP = peripapillary; VD = vessel density; MD = mean deviation; PSD = pattern standard deviation. Continuous values are presented as means ± standard deviations unless indicated otherwise.

**Table 2:** Comparisons of structural and functional parameters between the groups with higher and lower GC/IPL VD ratio.

Variables	High GC/IPL VD Ratio	Low GC/IPL VD Ratio	*p*-Value
	(*n* = 33)	(*n* = 33)	
Age, years	54.42 ± 11.63	53.03 ± 12.93	0.647
BCVA, decimals	0.95 ± 0.10	0.92 ± 0.14	0.190
Baseline IOP, mmHg	15.33 ± 2.52	15.34 ± 2.59	0.987
Mean IOP, mmHg	14.50 ± 2.72	13.53 ± 2.55	0.141
CCT, µm	535.91 ± 34.78	532.61 ± 36.50	0.715
Spherical equivalent, D	−2.00 ± 2.44	−2.12 ± 2.86	0.856
RNFL thickness, µm	90.91 ± 9.78	88.97 ± 12.48	0.485
GC/IPL thickness, µm	63.92 ± 5.33	62.81 ± 5.71	0.415
PP VD	45.67 ± 1.42	45.44 ± 1.16	0.457
PP VD ratio	0.82 ± 0.10	0.74 ± 0.14	**0.015**
RNFL VD (%)	41.44 ± 1.14	40.41 ± 1.17	**0.001**
RNFL VD ratio	0.83 ± 0.15	0.65 ± 0.13	**<0.001**
Disc hemorrhage, *n* (%)	6 (18.2%)	10 (30.3%)	0.251
Microvasculature dropout, *n* (%)	13 (39.4%)	17 (53.1%)	0.267
MD, dB	−1.84 ± 1.90	−2.95 ± 1.96	**0.022**
PSD, dB	3.63 ± 2.57	5.16 ± 2.96	**0.035**
Paracentral scotoma, *n* (%)	15 (45.5%)	23 (69.7%)	**0.046**
Diabetes mellitus, *n* (%)	2 (6.1%)	4 (12.1%)	0.392
Systemic hypertension, *n* (%)	1 (3.0%)	7 (21.2%)	**0.024**

BCVA = best corrected visual acuity; IOP = intraocular pressure; CCT = central cornea thickness; D = diopter; RNFL = retinal nerve fiber layer; GC/IPL = ganglion cell/inner plexiform layer; PP = peripapillary; VD = vessel density; MD = mean deviation; PSD = pattern standard deviation. Continuous values are presented as means ± standard deviations unless indicated otherwise. Independent sample t-tests and chi-squared tests were used. Statistically significant values are shown in boldface.

**Table 3:** Correlation coefficients for RNFL and GC/IPL VD ratio.

	RNFL VD Ratio		GC/IPL VD Ratio	
	R	*p*-Value *	R	*p*-Value *
Mean IOP, mmHg	0.081	0.52	0.14	0.262
CCT, µm	0.062	0.627	0.022	0.866
Spherical equivalent, D	0.078	0.541	−0.13	0.304
PP VD	0.125	0.318	0.128	0.306
PP VD ratio	0.341	**0.005**	0.451	**0.000**
RNFL thickness, µm	0.343	**0.005**	0.067	0.596
GC/IPL thickness, µm	0.409	**0.001**	0.034	0.783
MD, dB	0.284	**0.021**	0.243	**0.049**
PSD, dB	−0.519	**0.000**	−2.226	0.067

IOP = intraocular pressure; CCT = central cornea thickness; D = diopter; PP = peripapillary; VD = vessel density; RNFL = retinal nerve fiber layer; GC/IPL = ganglion cell/inner plexiform layer; MD = mean deviation; PSD = pattern standard deviation. R = correlation coefficient. * Pearson correlation analysis. Statistically significant values are indicated in boldface.

**Table 4:** Regression analysis of factors associated with the macular GC/IPL VD ratio.

Variables	Univariate Model		Multivariate Model	
	Odds Ratio, (95% CI)	*p*-Value	Odds Ratio (95% CI)	*p*-Value
Age, years	0.000, (−0.004 to 0.003)	0.815		
Baseline IOP, mmHg	−0.003, (−0.019 to 0.012)	0.677		
Mean IOP, mmHg	0.008, (−0.006 to 0.023)	0.262		
CCT, µm	9.810E-5, (−0.001 to 0.001)	0.866		
Spherical equivalent, D	−0.008, (−0.023 to 0.007)	0.304		
RNFL thickness, µm	0.001, (−0.003 to 0.004)	0.596		
GC/IPL thickness, µm	0.001, (−0.006 to 0.008)	0.783		
PP VD	0.016, (−0.015 to 0.046)	0.306		
RNFL VD	0.043, (0.013 to 0.072)	**0.005**	0.038, (0.007 to 0.069)	**0.017**
Disc hemorrhage	−0.043, (−0.134 to 0.048)	0.348		
Microvasculature dropout	−0.04, (−0.119 to 0.039)	0.319		
MD, dB	0.019, (0.000 to 0.038)	0.049	0.016, (−0.009 to 0.041)	0.2
PSD, dB	−0.013, (−0.026 to 0.001)	0.067	−0.002, (−0.020 to 0.016)	0.847
Paracentral scotoma	−0.076, (−0.153 to 0.001)	0.053	−0.07, (−0.092 to 0.078)	0.878
Diabetes mellitus	−0.005, (−0.191 to 0.080)	0.417		
Systemic hypertension	−0.145, (−0.260 to −0.031)	**0.014**	−0.124, (−0.235 to −0.012)	**0.03**

CI = confidence interval; IOP = intraocular pressure; CCT = central cornea thickness; D = diopter; RNFL = retinal nerve fiber layer; GC/IPL = ganglion cell/inner plexiform layer; PP = peripapillary; VD = vessel density; MD = mean deviation; PSD = pattern standard deviation. Statistically significant values are indicated in boldface.

**Table 5:** Regression analysis of the factors associated with the inferior RNFL VD to PP VD ratio.

Variables	Univariate Model		Multivariate Model	
	Odds Ratio, (95% CI)	*p*-Value	Odds Ratio, (95% CI)	*p*-Value
Age, years	−0.001, (−0.003 to 0.002)	0.645		
Baseline IOP, mmHg	0.007, (−0.004 to 0.018)	0.196		
Mean IOP, mmHg	0.002, (−0.008 to 0.013)	0.671		
CCT, µm	0.000, (−0.001 to 0.001)	0.54		
Spherical equivalent, D	0.006, (−0.004 to 0.017)	0.235		
RNFL thickness, µm	0.002, (0.000 to 0.005)	0.089	0.002, (−0.001 to 0.004)	0.13
GC/IPL thickness, µm	0.006, (0.001 to 0.010)	**0.021**	−0.001, (−0.006 to 0.005)	0.801
GC/IPL VD	0.002, (−0.014 to 0.019)	0.778		
Disc hemorrhage	−0.054, (−0.117 to 0.008)	0.087	−0.027, (−0.083 to 0.029)	0.338
Microvasculature dropout	−0.043, (−0.097 to 0.012)	0.125		
MD, dB	0.008, (−0.006 to 0.021)	0.265		
PSD, dB	−0.012, (−0.021 to −0.002)	**0.015**	−0.002, (−0.012 to 0.007)	0.605
Paracentral scotoma	−0.125, (−0.171 to −0.079)	**<0.001**	−0.115, (−0.167 to −0.064)	**<0.001**
Diabetes mellitus	0.004, (−0.092 to 0.099)	0.938		
Systemic hypertension	−0.015, (−0.099 to 0.069)	0.717		

CI = confidence interval; IOP = intraocular pressure; CCT = central cornea thickness; D = diopter; RNFL = retinal nerve fiber layer; GC/IPL = ganglion cell/inner plexiform layer; PP = peripapillary; VD = vessel density; MD = mean deviation; PSD = pattern standard deviation. Statistically significant values are indicated in boldface.

**Table 6:** Regression analysis of the factors associated with the inferior GC/IPL VD to PP VD ratio.

Variables	Univariate Model		Multivariate Model	
	Odds Ratio, (95% CI)	*p*-Value	Odds Ratio, (95% CI)	*p*-Value
Age, years	−0.001, (−0.003 to 0.002)	0.63		
Baseline IOP, mmHg	0.004, (−0.007 to 0.016)	0.466		
Mean IOP, mmHg	0.002, (−0.008 to 0.013)	0.671		
CCT, µm	0.000, (−0.001 to 0.001)	0.952		
Spherical equivalent, D	0.001, (−0.010 to 0.012)	0.88		
RNFL thickness, µm	−0.001, (−0.004 to 0.001)	0.39		
GC/IPL thickness, µm	−0.002, (−0.007 to 0.003)	0.507		
RNFL VD	0.014, (−0.009 to 0.036)	0.232	0.011, (−0.013 to 0.034)	0.365
Disc hemorrhage	0.004, (−0.063 to 0.070)	0.915		
Microvasculature dropout	−0.007, (−0.066 to 0.051)	0.801		
MD, dB	0.004, (−0.010 to 0.019)	0.543		
PSD, dB	0.003, (−0.007 to 0.013)	0.502		
Paracentral scotoma	−0.03, (−0.087 to 0.028)	0.308	0.002, (−0.058 to 0.061)	0.952
Diabetes mellitus	−0.029, (−0.128 to 0.070)	0.558		
Systemic hypertension	−0.142, (−0.222 to −0.061)	**0.001**	−0.138, (−0.211 to −0.055)	**0.001**

CI = confidence interval; IOP = intraocular pressure; CCT = central cornea thickness; D = diopter; RNFL = retinal nerve fiber layer; GC/IPL = ganglion cell/inner plexiform layer; VD = vessel density; MD = mean deviation; PSD = pattern standard deviation. Statistically significant values are indicated in boldface.

## Data Availability

Data may be provided upon reasonable request.
